# DNMT1/PKR double knockdowned HepG2 (HepG2-DP) cells have high hepatic function and differentiation ability

**DOI:** 10.1038/s41598-022-25777-z

**Published:** 2022-12-07

**Authors:** Rieko Tanaka-yachi, Kazuko Aizawa, Kie Shimizu, Hidenori Akutsu, Kazuaki Nakamura

**Affiliations:** 1 Department of Pharmacology, National Research Institute for Child Health and Development, Okura 2-10-1, Setagaya-Ku, Tokyo, 157-8535 Japan; 2 Faculty of Bioscience, Graduate School of Science and Engineering, Saitama University, Shimo-Okubo 255, Sakura-Ku, Saitama-Shi, Saitama, 338-8570 Japan; 3 Center for Regenerative Medicine, National Research Institute for Child Health and Development, Okura 2-10-1, Setagaya-Ku, Tokyo, 157-8535 Japan

**Keywords:** Cell biology, Drug discovery, Molecular biology

## Abstract

HepG2 cells are widely used as a human hepatocytes model, but their functions, including drug metabolism, are inferior to primary hepatocytes. We previously reported that the hepatic gene expressions in HepG2 cells were upregulated by treatment with zebularine, which is an inhibitor of DNA methylation, through the inhibition of both DNA methyltransferase 1 (DNMT1) and double-stranded RNA-dependent protein kinase (PKR). In this study, we established a new HepG2 cell subline, HepG2-DP cells, by stable double knockdown of DNMT1 and PKR and evaluated its function. Albumin production, expression of CYP1A2 genes, and accumulation of lipid droplets were increased in HepG2-DP cells compared with the original HepG2 cells. Comprehensive gene expression analysis of transcription factors revealed that the expression of important genes for hepatic function, such as HNF1β, HNF4α, ONECUT1, FOXA1, FOXA2, FOXA3, and various nuclear receptors, was upregulated in HepG2-DP cells. These results indicate that the newly established HepG2-DP cells are a highly functional hepatocyte cell line. In addition, we investigated whether HepG2-DP cells are able to mature by differentiation induction, since HepG2 cells are derived from hepatoblastoma. The gene expression of major CYPs and Phase II, III drug-metabolizing enzyme genes was significantly increased in HepG2-DP cells cultured in differentiation induction medium. These results suggest that HepG2-DP cells can be further matured by the induction of differentiation and could therefore be applied to studies of drug metabolism and pharmacokinetics.

## Introduction

The liver is essential for maintaining normal physiology and homeostasis of the body and is the primary site of metabolism for most drugs. In particular, hepatocytes play essential roles in metabolism, detoxification, bile acid synthesis, and protein synthesis. In drug discovery research, non-human hepatocytes are not suitable because the expression pattern of drug-metabolizing enzymes differs depending on the animal species^1^. Therefore, human hepatocytes are the most important research tool in preclinical research, such as drug metabolism and toxicity tests. However, due to the limited proliferative capacity and lot-to-lot differences of primary human hepatocytes (PHHs), the development of a proliferative and practical human hepatocyte cell model is needed.

Currently, there are approximately 40 different hepatic tumor cell lines, but the most commonly used are HepaRG, Huh7, SK-Hep-1, Hep3B, and HepG2, which are obtained from various tumors. Among the aforementioned cells, the HepG2 cell line has gained popularity due to its wide range of applications in scientific research^2^. HepG2 cells, which are liver cancer–derived cell lines, are widely used as a human hepatocytes model. However, their functions are decreased overall compared with PHHs^3–6^. The appearance of metabolites and toxicity in HepG2 cells does not reflect correct hepatic function.

Various efforts have been made to improve the hepatic function of HepG2 cells. For example, 3D culture of HepG2 cells enhances the hepatocyte-specific functions, including drug-metabolizing enzyme activities^7–9^. In contrast, Oshikata-Miyazaki et al. reported that the hepatic structure and functions of monolayered HepG2 cells can be induced within a day after oxygenation using a collagen vitrigel membrane^10^. Recently, a new subline of HepG2 cells, HepG2-NIAS, was reported. The liver-specific functions of HepG2-NIAS cells are rapidly enhanced by oxygenation culture^11^. A battery of HepG2-derived cell lines that individually express 14 cytochrome P450s (CYPs) (1A1, 1A2, 1B1, 2A6, 2B6, 2C8, 2C9, 2C18, 2C19, 2D6, 2E1, 3A4, 3A5, and 3A7) was also generated^12^.

In these attempts, we have previously reported that the exposure to zebularine enhances the hepatic function of HepG2 cells^13^. Zebularine (1-(β-d-ribofuranosyl)-1, 2-dihydropyrimidin-2-one), which is a nucleoside analog of cytidine, is a highly stable hydrophilic inhibitor of DNA methylation^14^. DNA methylation is an important process involved in many biological phenomena and its abnormalities are involved in the poor cell-specific function of cancer cells, including HepG2 cells. Many gene promoters have CpG-rich regions (CpG islands) and methylation of CpG islands is involved in transcriptional inactivation. DNA is methylated by a family of enzymes called DNA methyltransferases (DNMTs). The findings that many genes in HepG2 cells are turned on or upregulated though DNA methyltransferase 1 (DNMT1) inhibition by zebularine suggest that excessive methylation by DNMT1 is involved in the hypofunction of HepG2 cells. In addition. we have revealed that the hepatic gene expressions upregulated by zebularine were through the inhibition of double-stranded RNA-dependent protein kinase (PKR) as well as DNMT1 in HepG2 cells^13^. Thus, the induction of hepatic function by zebularine is expected to contribute to the application of HepG2 cells to drug metabolism research. However, due to concerns about drug interactions, drug-free culture systems are required in metabolic tests and toxicity tests.

To resolve this problem, in this study, we established a new HepG2 cell subline, HepG2-DP cells, by stable double knockdown of DNMT1 and PKR and evaluated its function. In addition, because HepG2 cells were derived from hepatoblastoma, we examined the differentiation potential to mature hepatocytes of HepG2-DP cells in this study.

## Results

### Establishment of HepG2-DP cells by DNMT1 and PKR knockdown

Original HepG2 cells express DNMT1 and PKR. We attempted the stable knockdown of both DNMT1 and PKR by retrovirus vector expressing shRNAs against both DNMT1 and PKR and found that these proteins were almost disappeared in the pre-cloning cell population in HepG2 cells (Fig. S1A). Pre-cloning knockdown HepG2 cells were cultured at low density and the colony was observed as shown in Fig. S1B. Cells were cloned by single colony picking and four clones with similar proliferative potential as the original HepG2 cells were selected as HepG2-DP cells (Fig. S1C). The expression level of DNMT1 almost disappeared but the expression of PKR remained at a low level in the four clones (Fig. S1D). Because HepG2-DP clone No.4 (DP-4) had the most reduced PKR expression (Fig. S1D) and the highest expression of CYP3A4, which is the most important molecular species involved in many drug metabolisms in humans, and ALB among four clones (Fig. S1E), we selected DP-4 as HepG2-DP and used it in the subsequent experiments in this study. The integration site of shRNA against DNMT1 and PKR in HepG2-DP cells was examined by whole-genome sequencing. We confirmed that the shRNA sequence was integrated on chromosome 5:95,809,436. Examination of the genome data of the National Library of Medicine (National Center for Biotechnology Information, USA) confirmed that no gene was present at this location.

**Figure 1:**
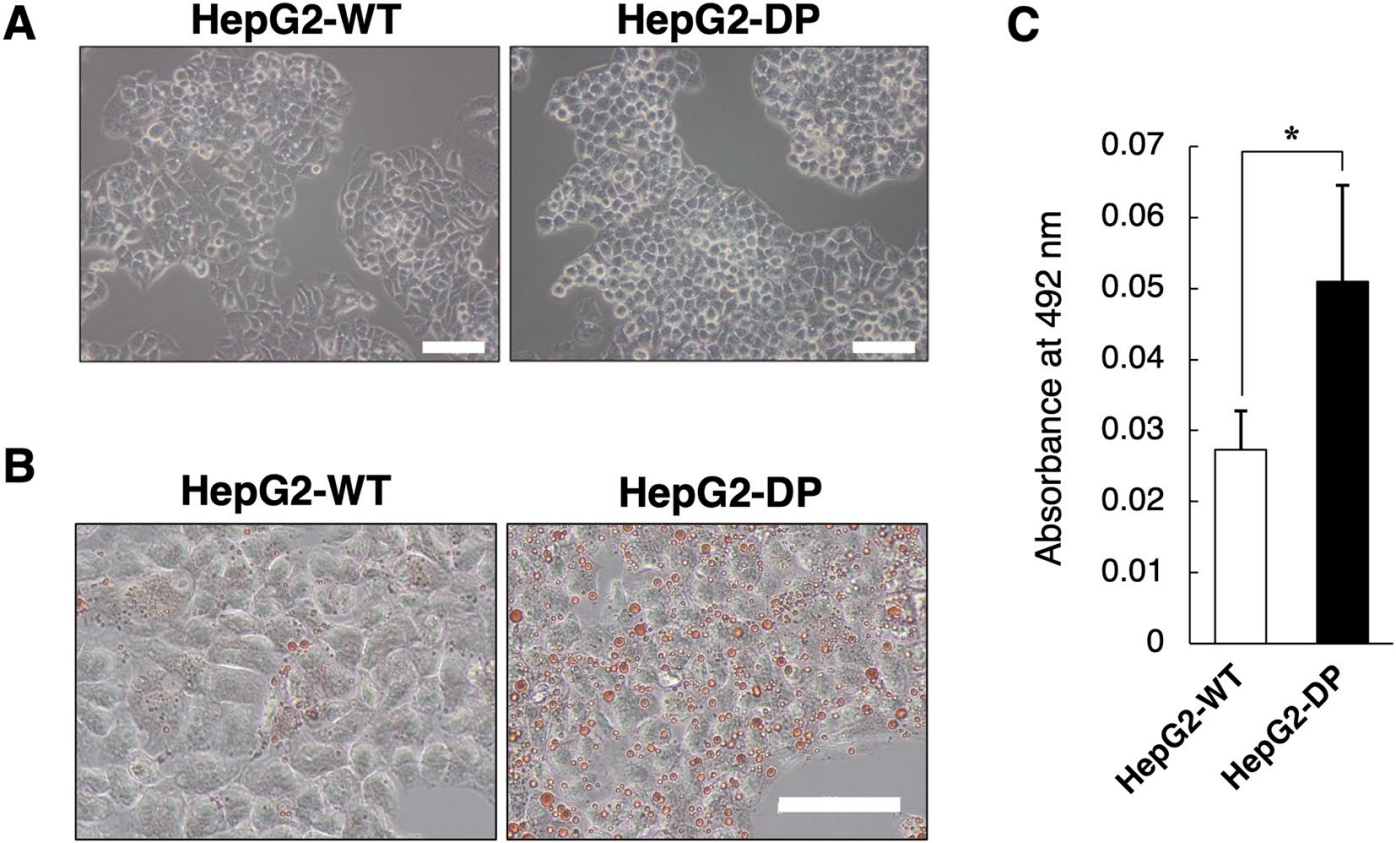
Morphology and lipid accumulation in HepG2-DP cells. (**A**) Phase-construct image of HepG2-WT and HepG2-DP cells. The cells were cultured for 72 h. Scale bar, 100 μm. (**B**) Oil-red O staining of HepG2-WT and HepD2-DP cells. The cells were cultured for 72 h and then fixed and stained with oil-red O. Scale bar, 40 μm. (**C**) Absorbance of oil red O. Absorbance at 492 nm was measured on oil red O–stained culture plates. The values are the mean ± standard deviation for three samples. Statistical analysis was performed by applying the unpaired *t* test (*p < 0.05).

The morphology of HepG2-DP cells was epithelial-like compared to the original HepG2 cells (HepG2-WT) and the cell–cell adhesion appeared enhanced (Fig. 1A). The gene expressions of intercellular adhesion factors were examined. The expression level of tight junction protein 1 (TJP1, also known as ZO-1), which acts as a tight junction adaptor protein that also regulates adherens junctions, was significantly increased in HepG2-DP cells compared with HepG2 cells, suggesting that cell–cell adhesions were enhanced in HepG2-DP cells (Fig. S2). In addition, the accumulation of lipid droplets, which is characteristic of hepatocytes, was confirmed and significantly increased in HepG2-DP cells (Fig. 1B,C). These results suggested that HepG2-DP cells have more hepatocyte characteristics than HepG2-WT.

### Hepatic gene expression profile and function of HepG2-DP cells

**Figure 2:**
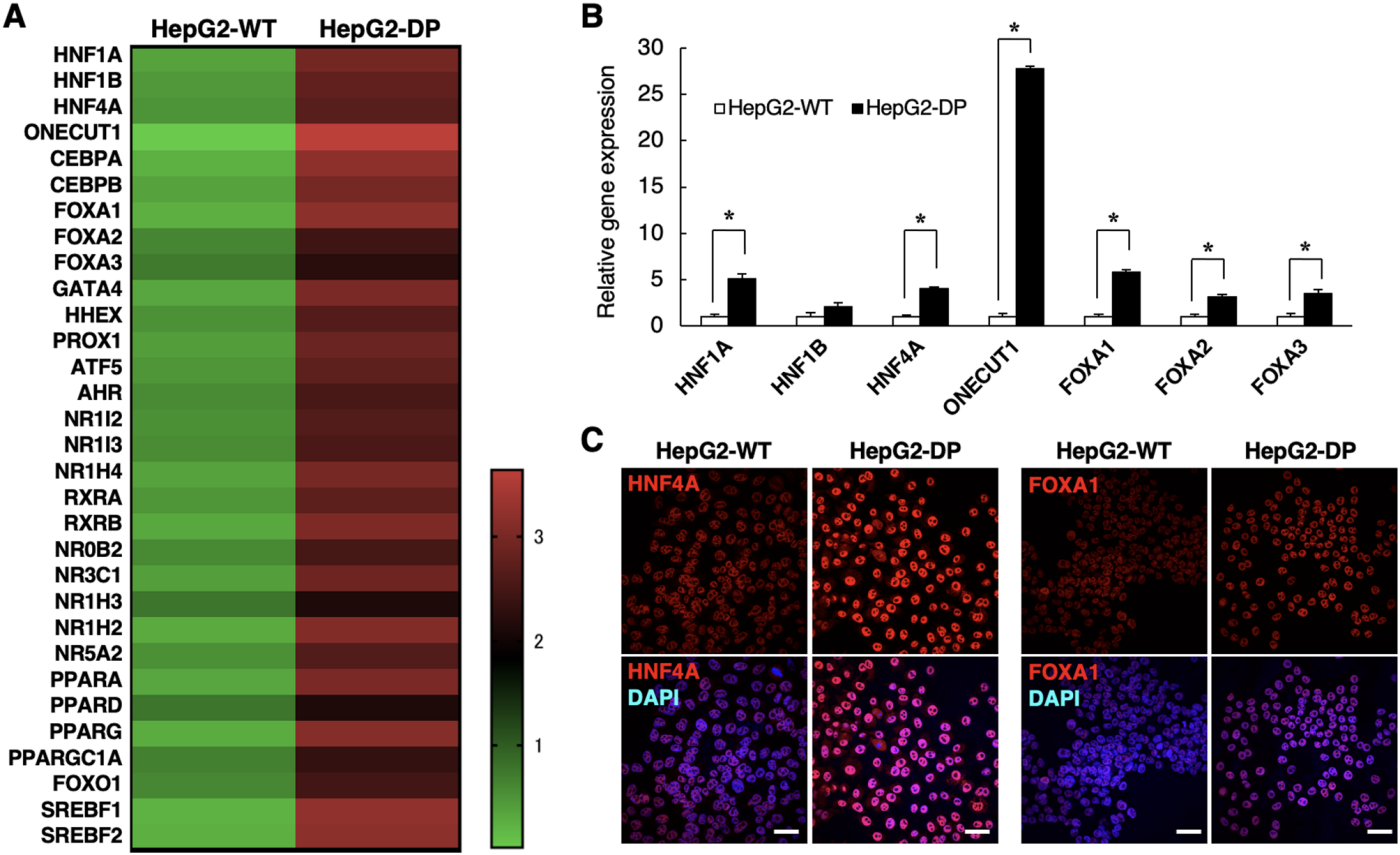
Gene expression profile of transcription factors. (**A**) Gene expression array for hepatic transcription factors. HepG2-WT and HepD2-DP cells were cultured for 72 h. The expression level was corrected by the average value, and the data was visualized as a heat map. (**B**) Gene expression analysis of transcription factors by quantitative polymerase chain reaction. The values are the mean ± standard deviation for three samples. Statistical analysis was performed by applying the unpaired *t* test (*p < 0.05). (**C**) Immunofluorescence staining for HNF4α and FOXA1. Cells were cultured for 72 h. Immunofluorescence staining was performed to detect transcriptional factors (in red) and observed by confocal microscopy. The cell nuclei were stained with DAPI (in blue). Scale bar, 40 μm.

**Figure 3:**
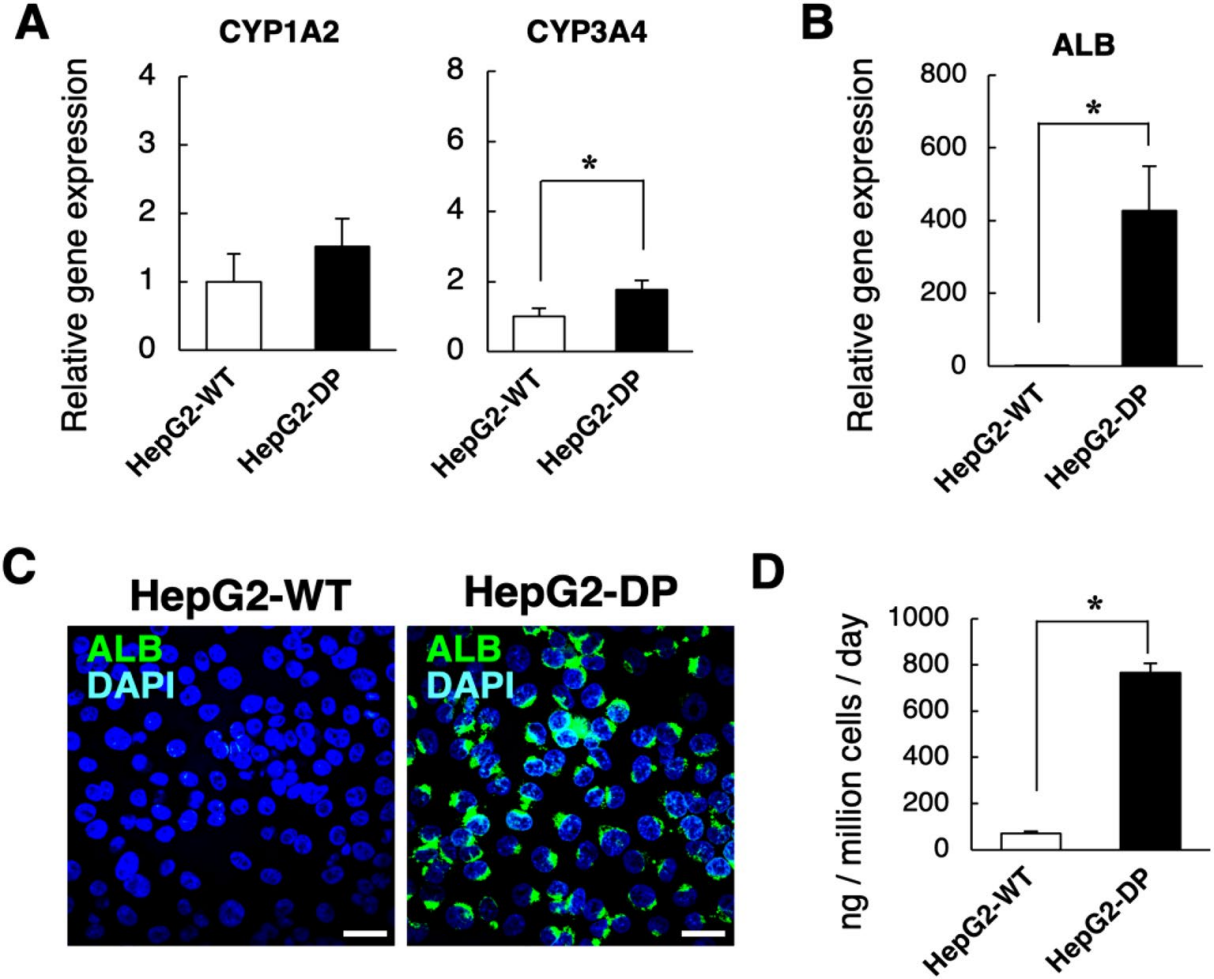
CYPs gene expression and albumin production in HepG2-DP cells. (**A**) Gene expression of CYP1A2 and CYP3A4. Cells were cultured for 72 h. Reverse transcription polymerase chain reaction (RT-PCR)-examined gene expression levels of CYP1A2 and CYP3A4. (**B**) Gene expression of albumin (ALB). Cells were cultured for 72 h. RT-PCR-examined gene expression levels of ALB. (**C**) Immunofluorescence staining for albumin. Cells were cultured for 72 h. Immunofluorescence staining was performed to detect albumin (in green) and observed by confocal microscopy. The cell nuclei were stained with DAPI (in blue). Scale bar, 40 μm. The exposure time of the fluorescence micrographs was adjusted to the ALB immunopositive signals of HepG2-DP cells, resulting in very weak ALB immunopositive signals in HepG2-WT at the same exposure time. (**D**) Albumin production of HepG2-WT and HepG2-DP cells. Albumin production per 24 h was quantified by enzyme-linked immunosorbent assay. The values are the mean ± standard deviation for three samples. Statistical analysis was performed by applying the *t* test (*p < 0.05).

To further investigate the characteristics of HepG2-DP cells, we analyzed the expression of the transcription factors involved in hepatic function using gene expression arrays. Many transcription factors tended to be induced in HepG2-DP cells compared with HepG2-WT cells (Fig. 2A). Among these transcription factors, we examined the expression level of seven transcription factors, which are important sets for hepatocyte differentiation and function, by qRT-PCR. Gene expression levels of HNFs, such as HNF1A, HNF4A, and ONECUT1 and FOXA1, 2, 3, were significantly higher in HepG2-DP cells than in HepG2-WT cells (Fig. 2B). In addition, fluorescent immunostaining of HNF4α and FOXA1 revealed their nuclear localization (Fig. 2C). These results suggest that suppression of both DNMT1 and PKR activates the expression of various transcription factors and affects hepatic functions, including metabolism, albumin secretion, and bile acid synthesis and secretion. Thus, we compared the gene expression of drug-metabolizing enzymes (CYP1A2 and 3A4) and albumin, which are indicators of hepatic function, between HepG2-WT and HepG2-DP cells. Although there was no significant difference in the CYP1A2 gene expression level between HepG2-WT and HepG2-DP cells, the gene expression of CYP3A4 of HepG2-DP cells was significantly increased compared to that of HepG2-WT (Fig. 3A). The gene expression level of albumin in HepG2-DP cells was increased more than 400-fold compared with that of HepG2-WT cells (Fig. 3B), and albumin was strongly stained near the nucleus by fluorescent immunostaining in HepG2-DP cells (Fig. 3C). The amount of secreted albumin from HepG2-DP cells to the medium increased approximately tenfold compared with HepG2-WT cells (Fig. 3D). We also performed gene expression analysis of alcohol dehydrogenase (ADH) and aldehyde dehydrogenases (ALDH), and an alcohol toxicity test to investigate the ability of alcohol metabolism and acetaldehyde-induced toxicity in HepG2-DP cells. Gene expression levels of ADH and ALDH were increased in HepG2-DP cells compared with HepG2-WT cells but were significantly lower than in PHHs (Fig. S3A). The degree of cell death by ethanol treatment in HepG2-DP cells was comparable to that in HepG2-WT cells (Fig. S3B). These results indicate that HepG2-DP cells have at least partial high hepatic function, including drug-metabolizing enzyme expression and albumin synthesis and secretory ability.

**Figure 4:**
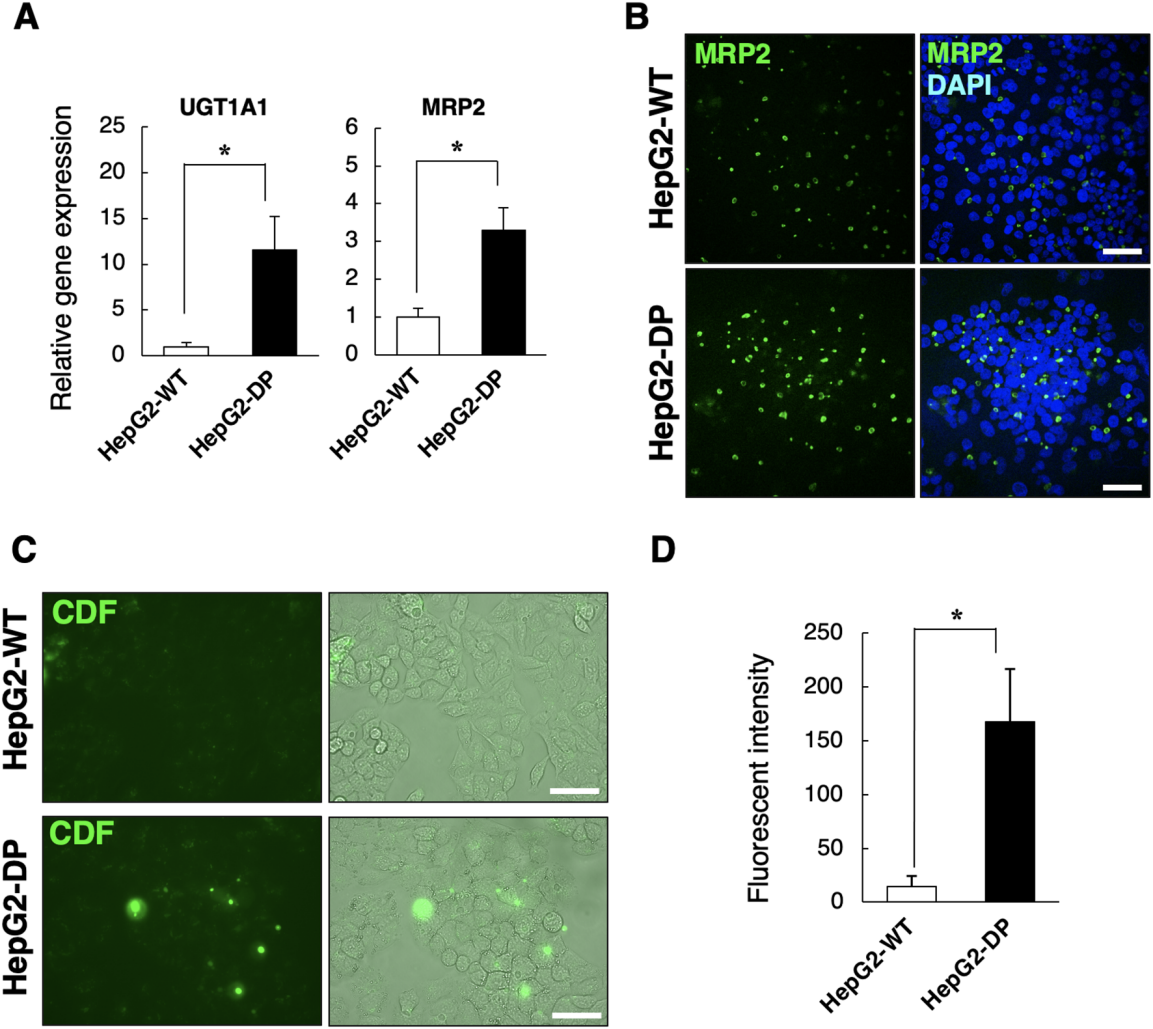
Bile acid conjugation and transport in HepG2-DP cells. (**A**) Gene expression of UGT1A1 and MRP2. Cells were cultured for 72 h. Reverse transcription polymerase chain reaction–examined gene expression levels of UGT1A1 and MRP2. (**B**) Immunofluorescence staining of MRP2. Cells were cultured for 72 h and then fixed and stained for MRP2 (in green). The cell nuclei were stained with DAPI (in blue). Scale bar, 40 μm. (**C**) MRP2 transporter activity assay. CDF were accumulated in the bile canaliculus in HepG2-DP cells (Arrows). Scale bar, 40 μm. (**D**) Fluorescence intensity of CDF. The fluorescence intensity of CDF (Ex. 488 nm/Em. 517 nm) accumulated in the bile canaliculi was measured. The values are the mean ± standard deviation for five samples. Statistical analysis was performed by applying the *t* test (*p < 0.05).

We further examined the hepatic function of HepG2-DP cells. The gene expression of uridine diphosphate glucuronosyltransferase1A1 (UGT1A2) and multidrug resistance–associated protein 2 (MRP2), which are, respectively, a bile acid conjugation enzyme and a bile acid transporter, were higher in HepG2-DP cells compared with HepG2-WT cells (Fig. 4A). It is known that MRP2 is localized in the bile canaliculi formed between hepatocytes^15,16^. Although fluorescent immunostaining for MRP2 revealed that MRP2 was localized at the bile canaliculi surface in both HepG2-WT and HepG2-DP cells (Fig. 4B), HepG2-DP cells showed more and stronger MRP2 immunopositive signals than HepG2-WT cells. We also examined the functional activity of MRP2. A cellular esterase converts 5-(and-6)-carboxy-2ʹ,7ʹ-dichlorofluorescein diacetate (CDFDA) into 5-(and-6)-carboxy-2ʹ,7ʹ-dichlorofluorescein (CDF). CDF is a substrate of MRP2 and is excreted into canaliculi, in which it accumulates. We observed that CDF was excreted to the extracellular domains after 30 min of exposure to CDFDA and accumulated in the bile canaliculus in HepG2-DP cells. In contrast, CDF accumulation in the bile canaliculus was not observed in HepG2-WT cells (Fig. 4C). The fluorescence intensity of CDF accumulated in the bile canaliculi in HepG2-DP cells was more than 10 times that of HepG2-WT cells (Fig. 4D). These results suggest that bile acid transport capacity was enhanced in HepG2-DP cells.

### Responsiveness of HepG2-DP cells to differentiation induction

**Figure 5:**
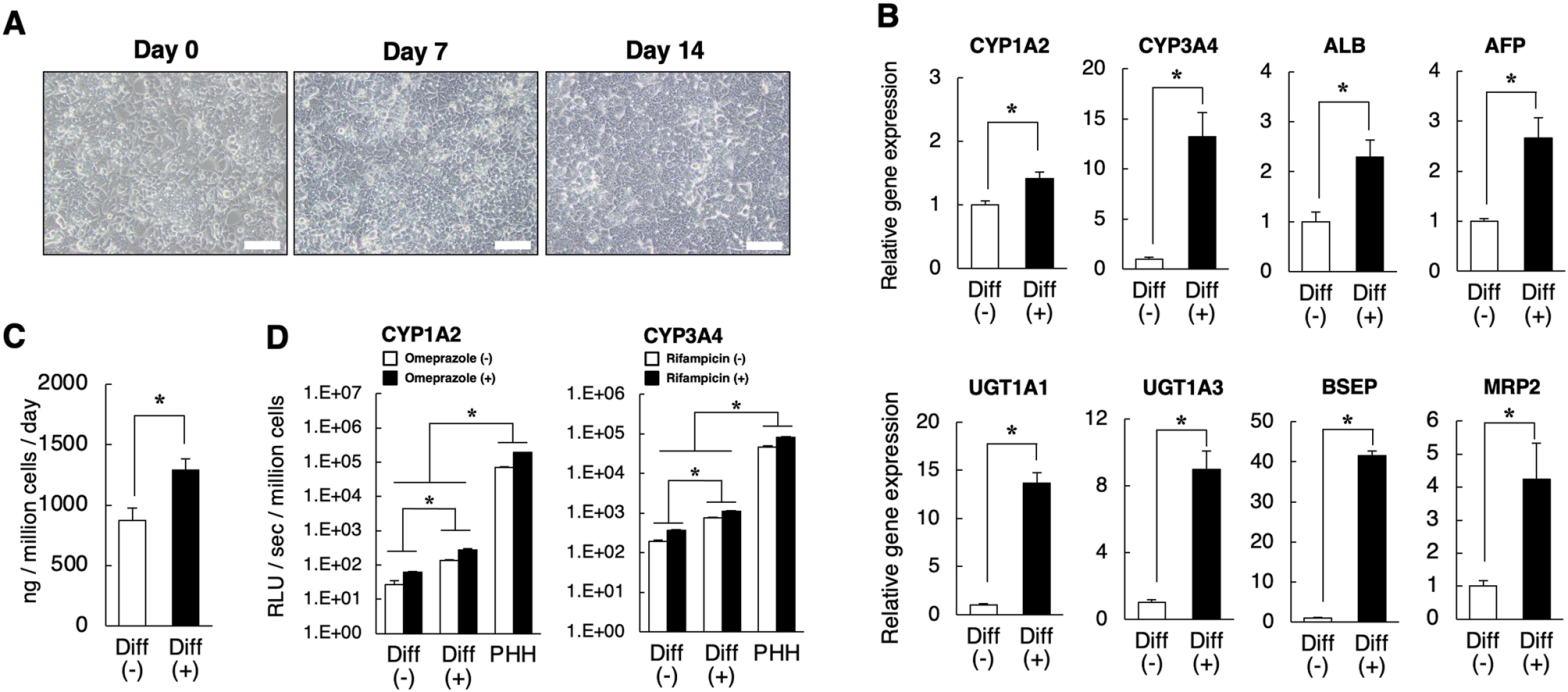
Hepatic function in differentiated HepG2-DP cells. (**A**) Phase-construct image of HepD2-DP cells cultured in differentiation medium. The cells were cultured in the differentiation medium for 0, 7, and 14 days. Scale bar, 100 μm. (**B**) Gene expression of CYPs, ALB, AFP, bile acid conjugation enzymes, and bile acid transporters in HepG2-DP cells. Cells were cultured for 72 h. Reverse transcription polymerase chain reaction–examined gene expression levels of CYP1A2, CYP3A4, ALB, AFP, UGT1A1, UGT1A3, BSEP, and MRP2. Diff (-) indicates the control and Diff ( +) indicates the differentiation condition. (**C**) Albumin production of differentiated cells. Albumin production per 24 h was quantified by enzyme-linked immunosorbent assay. D) CYP1A2 and CYP3A4 activity in differentiated cells. HepG2-DP cells were cultured in the differentiation medium for 14 days. HepG2-WT and PHHs were cultured for 48 h and then treated with the indicated inducers for 24 h. CYPs activities were examined using the P450-Glo assay kit. White bars indicate the no CYP inducer condition and black bars indicate the CYP inducer condition. The values are the mean ± standard deviation for three samples. Statistical analysis was performed by applying the *t* test (*p < 0.05).

As described above, HepG2-DP cells showed at least some enhancement in hepatic function compared to HepG2-WT cells. However, these functions were not considered sufficiently high. Based on the previous report, which demonstrated that the origin of HepG2 cells is hepatoblastoma, we next investigated whether HepG2-DP cells have the ability to differentiate into mature hepatocytes by culturing them in differentiation medium. HepG2-WT or HepG2-DP cells were cultured for 14 days in a differentiation medium. HepG2-WT cells continued to proliferate during the differentiation culture period and reached an overconfluent state (Fig. S4A) whereas HepG2-DP cells remained monolayered after induction of differentiation (Fig. 5A). These results suggest that HepG2-DP cells responded to the differentiation condition and transferred from the proliferation phase to the differentiation phase. In fact, under the differentiation condition, the gene expression of CYP1A2, CYP3A4, and ALB was significantly increased in HepG2-DP cells (Fig. 5B). Unexpectedly, the gene expression of α-fetoprotein (AFP), a marker of immature hepatocytes, was slightly increased by differentiation induction. Moreover, the gene expression of bile acid conjugation enzymes (UGT1A1 and UGT1A3) and bile acid transporters (BSEP and MRP2) were higher in differentiated HepG2-DP cells compared with undifferentiated HepG2-DP cells (Fig. 5B). This increase occurred not only in HepG2-DP cells but also in HepG2-WT, however, the response of HepG2-DP cells to the induction of differentiation was sufficiently higher than that of HepG2-WT (Fig. S4B). Albumin production in HepG2-DP cells was significantly increased by the induction of differentiation (Fig. 5C). In contrast, in HepG2-WT, albumin production decreased by the induction of differentiation for 14 days (Fig. S4C). This result suggests that the original HepG2 cells do not respond to differentiation induction, and that overconfluent culture has an undesirable effect. We also investigated CYPs activity and the responsiveness to CYP inducers in HepG2-DP cells. As previously reported^17^, original HepG2 cells do not respond to CYP inducers such as omeprazole and rifampicin (Fig. S4D). HepG2-DP cells showed high CYP activity in response to these inducers and, furthermore, differentiation-induced HepG2-DP cells showed higher drug metabolism activity induction than those without differentiation (Fig. 5D). The CYPs activity of differentiation-induced HepG2-DP cells was approximately 1/100 to 1/1000 of that of PHHs, but the rate of induction by CYP inducers was comparable to that of PHHs. These results suggest that HepG2-DP cells have the ability to differentiate into mature hepatocytes by culture in a differentiation-inducing medium.

## Discussion

In this study, we modified HepG2 cells with double knockdown of DNMT1 and PKR and established a new HepG2 cell subline: HepG2-DP cells. Our research team previously reported that the hepatic gene expressions were upregulated through the inhibition of both DNMT1 and PKR by zebularine^9^. DNA hypermethylation caused by elevated DNMT expression has been reported in many cancer cells. DNA methylation suppresses the expression of various functional genes and interferes with the original function of the cell^18–20^. PKR is a kinase whose activity is enhanced in cancer cells, and phosphorylation of eIF2a occurring downstream of PKR signaling negatively regulates transcription and protein synthesis^21,22^. Furthermore, JNK1 activated by PKR has been reported to be involved in histone H3 methylation and plays important roles in the development of human hepatocellular carcinoma through epigenetic mechanisms^23^. Taken together, these intracellular pathways, under the suppression of DNMT1 and PKR, could enhance the expression of many transcriptional factors, resulting in hepatic functional genes activation in HepG2-DP cells.

The HepG2-DP cells were generated by incorporating shRNA sequences into the genome of HepG2 cells, and there are no genes that regulate cell proliferation in the shRNA-incorporated genomic site. Therefore, although it is necessary to verify whether the function of HepG2-DP cells is maintained by long-term culture (multiple passages), HepG2-DP cells are expected to exhibit infinite proliferation ability similar to HepG2 cells while maintaining their function.

HepG2 cells are derived from hepatoblastoma. They have high migration ability and lose their characteristics as epithelial cells due to the epithelial–mesenchymal transition (EMT) that occurs in cancer cells. Changes in cell properties due to the EMT reduce the characteristic function of hepatocytes. Ruoß et al. reported that the suppression of DNA hypermethylation suppresses the EMT in HepG2 cells^24^. HepG2-DP cells exhibited a morphology similar to epithelial-like cells and the gene expression of tight junction protein. Gene expression of epithelial cell markers such as α-catenin and E-cadherin also tended to increase in HepG2-DP cells. In other words, HepG2-DP cells may have acquired epithelial properties such as intercellular adhesion, which is an intrinsic property of hepatocytes, resulting in increased hepatic function.

Gene expression array for hepatic transcription factors revealed that HepG2-DP cells showed an increase in many transcription factor genes. The increased gene expression of transcription factors regulating lipid synthesis, such as PPARα, PPARγ, and SREBFs^25^, may be involved in the formation of lipid droplets in HepG2-DP cells. Albumin production was significantly increased in HepG2-DP cells. This result is considered to be due to the increased gene expression of HNF1 and C/EBP that activate the transcription of the albumin gene^26^ in HepG2-DP cells. The increased expression of various transcription factors may also affect the expression of CYPs, phase II enzymes, and phase III transporters. Rifampicin and omeprazole induce CYPs activity by binding to PXR and AhR, respectively^27^. These nuclear receptors were highly expressed in HepG2-DP cells, and CYP activity was induced in response to rifampicin and omeprazole. Improving drug responsiveness is an important issue in drug metabolism testing applications, and HepG2-DP cells may solve the problems of HepG2 cells.

In this study, the degree of cell death by ethanol treatment in HepG2-DP cells was comparable to that in HepG2 cells. The biological response to ethanol is regulated by the processes of acetaldehyde production by ADH, CYP2E1, and catalase, and its metabolism to acetic acid by ALDH. The mechanism of alcohol-induced hepatocellular damage is thought to involve the redox shift associated with ethanol metabolism, toxicity of acetaldehyde, and increased oxidative stress due to reactive oxygen species (ROS) production^28^. When the results in Fig. S3A are analyzed by unpaired *t* test between HepG2-WT cells and HepG2-DP cells, ADH and ALDH gene expressions significantly increased in HepG2-DP cells compared with HepG2 cells (*p* = 0.0069 and *p* = 0.0037, respectively). Therefore, since both ADH and ALDH gene expression increases are comparable in HepG2-DP cells, it is possible that no difference in alcohol toxicity was observed between HepG2-WT cells and HepG2-DP cells as a result of the simultaneous increased production of acetaldehyde and its metabolism. However, the increase of ADH and ALDH gene expressions in HepG2-DP cells could expand the field of research such as alcohol metabolism using cell lines.

The HepG2 cell line was originally established by Aden et al. in 1979^29^, and López-Terrada et al. demonstrated that HepG2 cells were derived from hepatoblastoma by array comparative genomic hybridization analysis^30^. HepG2 cells cultured in a three-dimensional in vitro model using extracellular matrix–based hydrogel for long-term culture stop proliferating, self-organize, and differentiate to form multiple polarized spheroids^31^. Li et al. reported that co-culture with mouse embryonic hepatocytes promotes the maturation of HepG2, and that upregulation of HNF4α is an important mechanism^32^. Thus, it is possible that HepG2 cells have strong properties as hepatic progenitor cells and consequently have low function as mature hepatocytes. The original HepG2 cells lacked a transcription factor essential for hepatic differentiation^5,6^ and therefore did not respond to differentiation induction under the conditions in the present study. An important set of transcription factors required for hepatocyte differentiation (a combination of HNF4α and FOXA1, 2, 3) has been reported by Suzuki et al.^33^. Upregulation of these transcription factors in HepG2-DP cells may contribute to improved responsiveness to differentiation induction. Coinstantaneously, gene expression of HNF1α, HNF1β, and ONECUT1 (HNF6), which are important in the differentiation of biliary epithelial cells, were also significantly higher in HepG2-DP, suggesting that HepG2-DP cells regained properties similar to those of normal hepatoblasts. Optimization of culture conditions may also achieve differentiation of HepG2-DP cells into biliary epithelial cells. Further research is needed to establish the necessary factors for completely HepG2 differentiation and maturation.

Knockdown of DNMT1 and PKR also had a significant effect on bile acid excretion. The bile canaliculus structure is also observed in the original HepG2 cells, but the gene expression level of bile acid–conjugating enzymes and transporters is low, resulting in inadequate synthetic and transport capacity. Differentiated HepG2-DP cells showed high UGT1A1, 1A3, BSEP, and MRP2 gene expression levels. These results suggest that HepG2-DP cells can be used to study drug and bile acid excretion as well as metabolism in Phase I.

To summarize, we established a highly functional new HepG2 cell subline, HepG2-DP cells, by double knocking down DNMT1 and PKR. HepG2-DP cells simultaneously showed high responsiveness to the induction of differentiation, and the induction of differentiation further enhanced the functionality of HepG2-DP cells. These results indicate that differentiated HepG2-DP cells are a highly functional hepatocytes model and can be applied to drug metabolism or pharmacokinetic study.

## Methods

### Cell culture and differentiation

The HEK293 (JCRB9068) cells using in retrovirus production were purchased from the Health Science Research Resources Bank (Japan Health Sciences Foundation, Osaka, Japan). Cells were maintained at 37 °C with 5% CO_2_ in Dulbecco’s modified Eagle’s medium (DMEM) containing 10% fetal bovine serum, penicillin (100 U/mL), and streptomycin (100 µg/mL). Phase contrast cell images were obtained using an inverted microscope (CKX41, Olympus Corporation, Tokyo, Japan).

HepG2 cells (JCRB1054) were purchased from the Health Science Research Resources Bank (Japan Health Sciences Foundation). The source of the HepG2 cell line was authenticated by short tandem repeat (STR) analysis (Promega K.K., Tokyo, Japan). HepG2 cells were cultured at 37 °C with 5% CO_2_ in DMEM containing 10% fetal bovine serum, penicillin (100 U/mL), and streptomycin (100 mg/mL). For differentiation and maturation, HepG2 cells were cultured in DMEM supplemented with 10% fetal bovine serum, penicillin (100 U/mL), streptomycin (100 μg/mL), HGF (20 ng/mL), oncostatin M (20 ng/mL), and dexamethasone (0.1 mM) for 14 days.

Cryopreserved human hepatocytes (lot: BHum16059) were purchased from Cytes Biotechnologies S.L (Barcelona, Spain). PHHs were seeded in plating medium (MP100, LONZA K.K., Tokyo, Japan) and incubated for 4 h. Next, they were maintained in hepatocyte culture medium (HCM, LONZA K.K., Tokyo, Japan).

### Construction of retroviral vector and virus production

Small interfering RNA (siRNA)-expressing retrovirus vector was constructed using a pSINsi-DK I vector (TAKARA Bio Inc., Shiga, Japan). The oligo DNAs were synthesized as follows: shDNMT1; 5′-GATCCGTGAGTGGAAATTAAGACTTTATGTACTGTGAAGCCACAGATGGGTACATAAAGTCTTAATTTCCACTCACTTTTTTAT-3′ and 5′-CGATAAAAAAGTGAGTGGAAATTAAGACTTTATGTACCCATCTGTGGCTTCACAGTACATAAAGTCTTAATTTCCACTCACG -3′, shPKR; 5′-CTAGAGGCAAGACTATGGAAAGGAAGTGGACGTGTGCTGTCCGTCCACTTCCTTTCCATAGTCTTGCCTTTTTTCCTGCA-3′, and 5′-GGAAAAAAGGCAAGACTATGGAAAGGAAGTGGACGGACAGCACACGTCCACTTCCTTTCCATAGTCTTGCCT-3′.

The annealed oligo DNA, DK I Promoter Cassette, and linear pSINsi-DK I vector were ligated and transformed into competent cells according to the manufacturer’s instructions. The extracted vector was transfected into HEK293 cells with pGP vector and pE-Ampho vector (TAKARA Bio Inc.) using Lipofectamine 2000 (Thermo Fisher Scientific K.K., Tokyo, Japan). Culture supernatant 48 h after transfection was collected and concentrated using a Retro-Concentin™ retroviral concentration reagent (System Biosciences, LLC, Palo Alto, CA, USA). The titer of concentrated retrovirus was measured by a retrovirus titer set for real-time polymerase chain reaction (PCR) (TAKARA Bio Inc.) and cryopreserved at − 80 °C until use.

### Retroviral transduction

Viral transduction was performed on RetroNectin^®^-coated plates according to the manufacturer’s instructions (TAKARA Bio Inc.). A total of 0.5 mL of concentrated retrovirus supernatant was loaded on RetroNectin^®^-coated 24-well plates and incubated in a 5% CO_2_ incubator to promote binding of the virus particles with RetroNectin reagent. After 4 h, the supernatant was discarded, and the plate was washed with phosphate-buffered saline (PBS). Next, HepG2 cells were seeded at a density of 8 × 10^4^/cm^2^ and then cultured. After 48 h, cells were selectively cultured in medium containing 300 mg/mL G418 sulfate (Thermo Fisher Scientific K.K.). When the cell culture reached 80% confluency, cells were detached with 0.25% trypsin then subcultured on a 35 mm dish. Cells were cloned by single colony picking, and we named these clones HepG2-DP. The integration site of the shRNA in HepG2-DP cells was examined by whole-genome sequencing (NovaSeq 6000, Macrogen Japan Corp., Tokyo, Japan).

### Antibodies

Mouse ALB-FITC antibody (A80-229F) and rabbit anti-MRP2/ABCC2 antibody (R269) were purchased from Abcam PLC (Cambridge, UK). Goat anti-albumin antibody (A80-129A) was purchased from Bethyl Laboratories, Inc. (Montgomery, TX, USA). Rabbit anti-b-tubulin antibody (#2146) was purchased from CST Japan K.K. (Chiba, Japan). The concentration used in immunofluorescence staining and Western blot analysis was in accordance with the recommended protocol for each antibody.

### Oil-red O staining

Cultured cells were washed with PBS and fixed with 4% paraformaldehyde phosphate buffer solution (FUJIFILM Wako Pure Chemical Corporation, Osaka, Japan) at room temperature for 15 min. The fixed cells were then stained for 30 min at room temperature with a filtered 0.3% oil-red O/60% isopropanol solution, washed with distilled water, and observed using an inverted microscope (CKX41, Olympus Corporation). The staining intensity of oil-red O was quantified by measuring the absorbance at 492 nm using a Cytation-5 cell imaging multimode reader (Agilent Biotek, Tokyo, Japan).

### Immunofluorescence staining

Cultured cells were washed and fixed with 4% paraformaldehyde phosphate buffer solution at room temperature for 15 min. The fixed cells were permeabilized and blocked with PBS containing 1% horse serum and 0.4% Triton-X 100 for 45 min. This was followed by incubation with primary antibodies for 2 h, incubation with secondary antibodies conjugated to Alexa Fluor 488 (Thermo Fisher Scientific, Inc.) for 1 h, and then washing three times with PBS. Next, the cells were counterstained with DAPI and mounted. Images were obtained using a laser scanning microscope FV1000 (Olympus Corporation).

### Immunoblotting analysis

Total protein was extracted from cells using RIPA buffer (CST Japan K.K). Proteins were separated in a 4% to 20% Mini-PROTEAN TGX Gel (Bio-Rad Laboratories, Inc., Hercules, CA, USA) and then transferred to polyvinylidene difluoride membranes. The membranes were blocked for 5 min using EveryBlot blocking buffer (Bio-Rad Laboratories, Inc.) and then incubated overnight with primary antibodies and then with secondary antibodies conjugated to Alexa Fluor 680 (Thermo Fisher Scientific, Inc.) for 1 h. The fluorescence was visualized using a FUSION chemiluminescence imaging system (Vilber-Lourmat, Collégien, France).

### Measurement of gene expression by real-time PCR

Total RNA was extracted using an RNeasy mini kit (QIAGEN K.K., Manchester, UK). RNA quantity and purity were determined by measuring the absorbance at 258/280 nm. Total RNA was reverse-transcribed into cDNA using a high-capacity RNA-to-cDNA kit (Thermo Fisher Scientific, Inc.) according to the manufacturer’s protocol. The QuantStudio 12 K flex real-time PCR system (Thermo Fisher Scientific K.K.) and TaqMan^®^ Gene expression assays were used to analyze the gene expression levels according to the manufacturer’s instructions. GAPDH was used as an internal control. The assay IDs of the primer/probe mixtures in the TaqMan gene expression assays are shown in the supplemental information (Table S1). The relative ratio of each gene expression was evaluated using the comparative threshold cycle (Ct) method.

### Measurement of albumin by enzyme-linked immunosorbent assay

The cells were cultured for 72 h with medium exchange every 24 h. Albumin in the culture supernatant was measured using an enzyme-linked immunosorbent assay (ELISA) starter accessory kit and anti-albumin antibody (Bethyl Laboratories, Inc.) according to the manufacturer’s protocol. Albumin production was calculated per 24 h and corrected for cell number.

### MRP2 transporter activity

Cells were seeded at a density of 2 × 10^4^/cm^2^ and cultured for 72 h. Cultured cells were incubated with 10 µM 5-(and-6)-carboxy-2ʹ,7ʹ-dichlorofluorescein diacetate (CDFDA) for 30 min. The cells were then washed with PBS and incubated in culture medium for 2 h. Fluorescence images were obtained using the EVOS M7000 imaging system (Thermo Fisher Scientific K.K.). The fluorescence intensity of CDF (Ex. 488 nm / Em. 517 nm) accumulated in the bile canaliculi was measured by using Cytation-5 cell imaging multimode reader (Agilent Biotek Inc., Winooski, VT, USA).

### PCR array-based gene expression measurement

Extracted total RNA was reverse transcribed into cDNA as mentioned above. The array plate for the analysis of transcription factors was designed using a TaqMan^®^Gene expression array 96-well fast plate as a template (Thermo Fisher Scientific, Inc.). GAPDH was used as an internal control. The expression level was corrected by the average value, and the data was visualized as a heat map using GraphPad Prism (GraphPad Software).

### Measurement of CYP enzyme activities

The CYP1A2, 2C9, and 3A4 enzyme activities were determined using a P450-Glo assay kit (Promega K.K.). Prior to the assay, cells were treated with a CYP inducer (omeprazole or rifampicin) for 24 h. Cells were incubated in the assay medium containing luciferin-IPA for 1 h. The culture medium was transferred to 96-well plates, followed by the addition of the detection reagent. The plates were incubated for 20 min at room temperature, and then the luminescence intensity was measured using a microplate reader Cytation-5 cell imaging multimode reader (Biotek Instrument Inc., Winooski, VT, USA).

### Alcohol toxicity test

Cells were seeded at a density of 2 × 10^4^/cm^2^ and cultured for 72 h. To evaluate the toxicity of alcohol metabolites, acetaldehyde, cells were treated with each concentration of ethanol for 24 h. Cell viability was measured with a Vi-Cell XR cell viability analyzer system (Beckman Coulter Life Sciences K.K., Tokyo, Japan).

### Statistical analysis

All data are expressed as the mean ± standard deviation (SD). Statistical analyses were performed using unpaired *t* test or one-way factorial analysis of variance (ANOVA), followed by post hoc analysis and Tukey’s multiple comparison test. Differences were statistically significant at *p* < 0.05.

## Supplementary Information

Supplementary Information.

## Data Availability

The datasets analyzed during the current study are available from the corresponding author on reasonable request.
