# Supplementary material for: DNMT1/PKR double knockdowned HepG2 (HepG2-DP) cells have high hepatic function and differentiation ability
Source: Sci Rep. 2022 Dec 7;12:21173. doi: 10.1038/s41598-022-25777-z (PMC9729623; doi:10.1038/s41598-022-25777-z)
Supplement: Supplementary file 1 — Supplementary Information. [file 41598_2022_25777_MOESM1_ESM.docx]

**Figure S1**


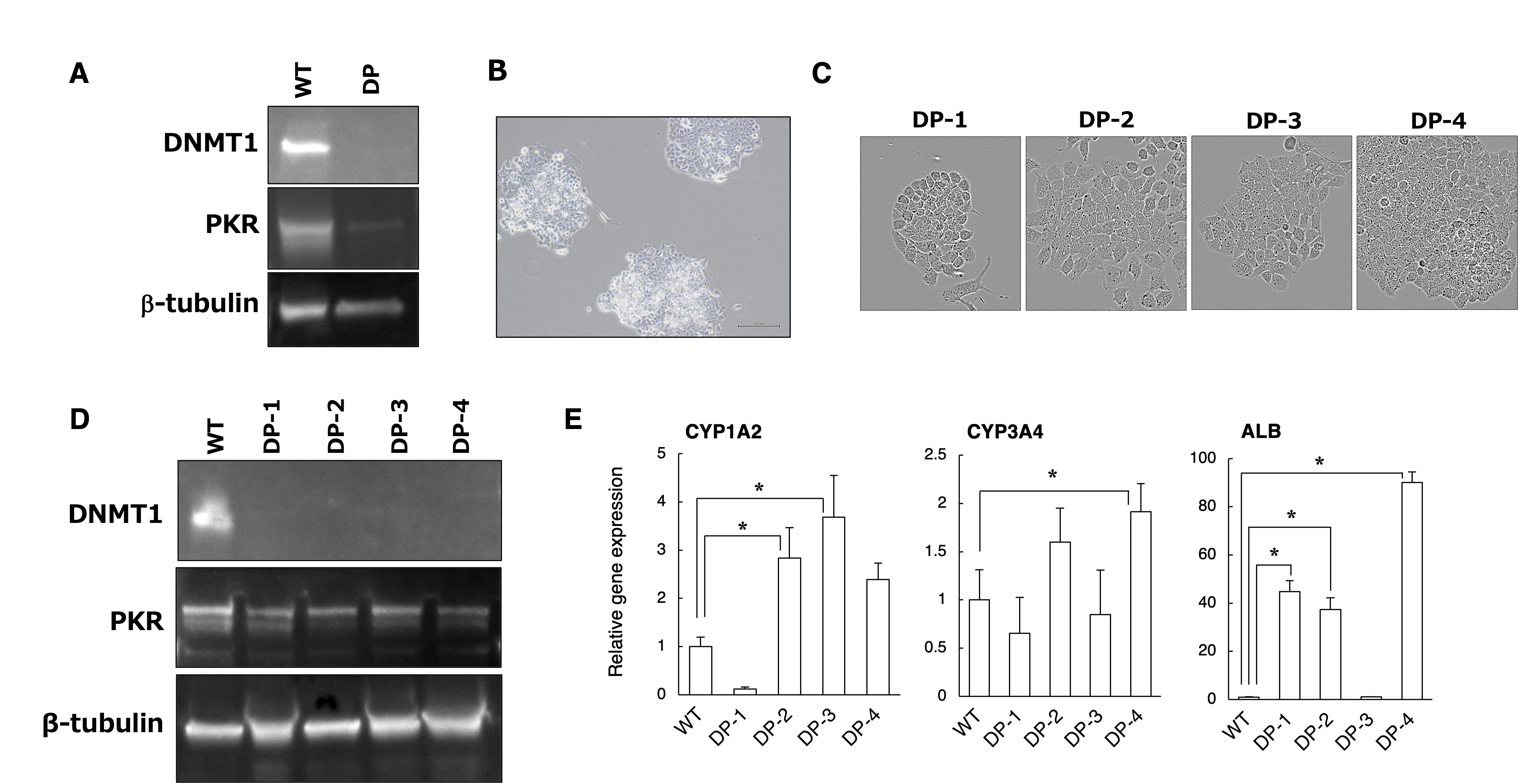


**Figure S1. Establishment of HepG2-DP cells.**

A) Protein expression of DNMT1 and PKR in HepG2-DP cells before cloning. The cells were harvested, and Western blot analysis was performed to measure DNMT1 and PKR levels. B, C) Phase-construct image of HepG2-DP colonies. Cells were cloned by single colony picking and numbering. D) Protein expression of DNMT1 and PKR in HepG2-DP clones. The cells were harvested, and Western blot analysis was performed to measure DNMT1 and PKR levels. E) Gene expression of CYP1A2, CYP3A4, and albumin. Cells were cultured for 72 h. Reverse transcription polymerase chain reaction (RT-PCR)-examined gene expression levels of CYP1A2, CYP3A4, and albumin. The values are the mean ± standard deviation for three samples. Statistical analysis was performed by applying the *t*-test (**p*< 0.05). According to molecular weight, the PVDF membrane was cut prior to hybridization with antibodies and original blots of A and D are presented in Figure S5.

**Figure S2**


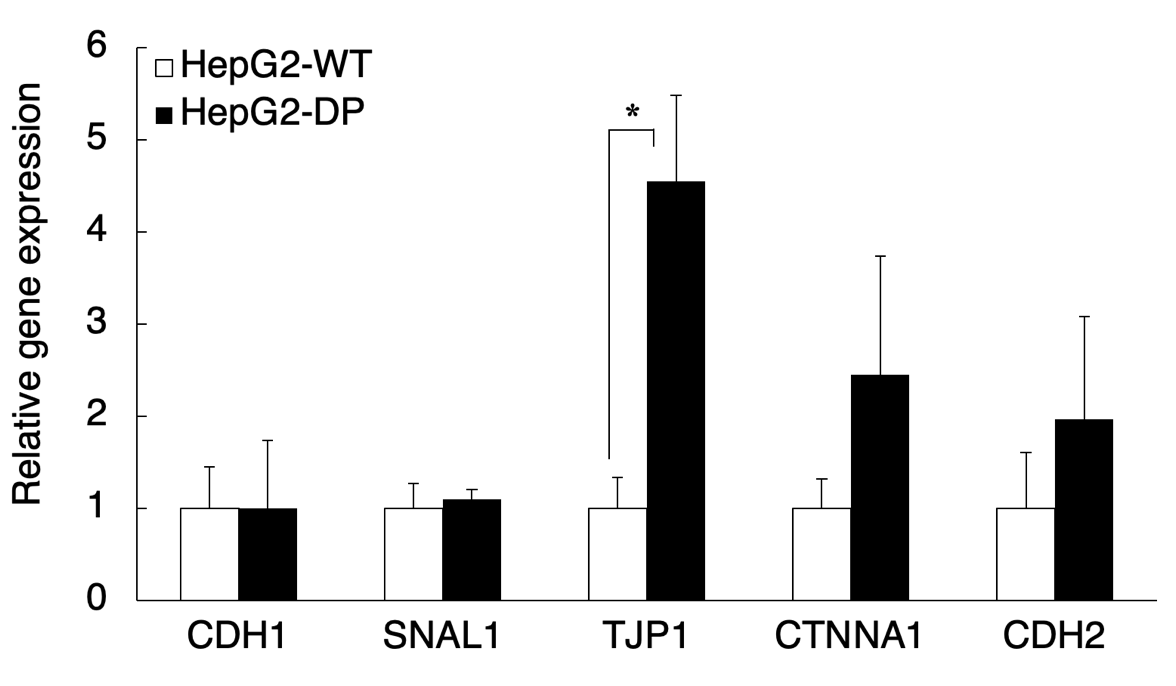


**Figure S2.** **Gene expression of cell–cell adhesion factors.**

Gene expression analysis for cell–cell adhesion factors. HepG2-WT and HepG2-DP cells were seeded and cultured for 72 h. Reverse transcription polymerase chain reaction (RT-PCR)-examined gene expression levels of cell–cell adhesion factors. The values are the mean ± standard deviation for three samples. Statistical analysis was performed by applying the *t*-test (**p*< 0.05).

**Figure S3**

**
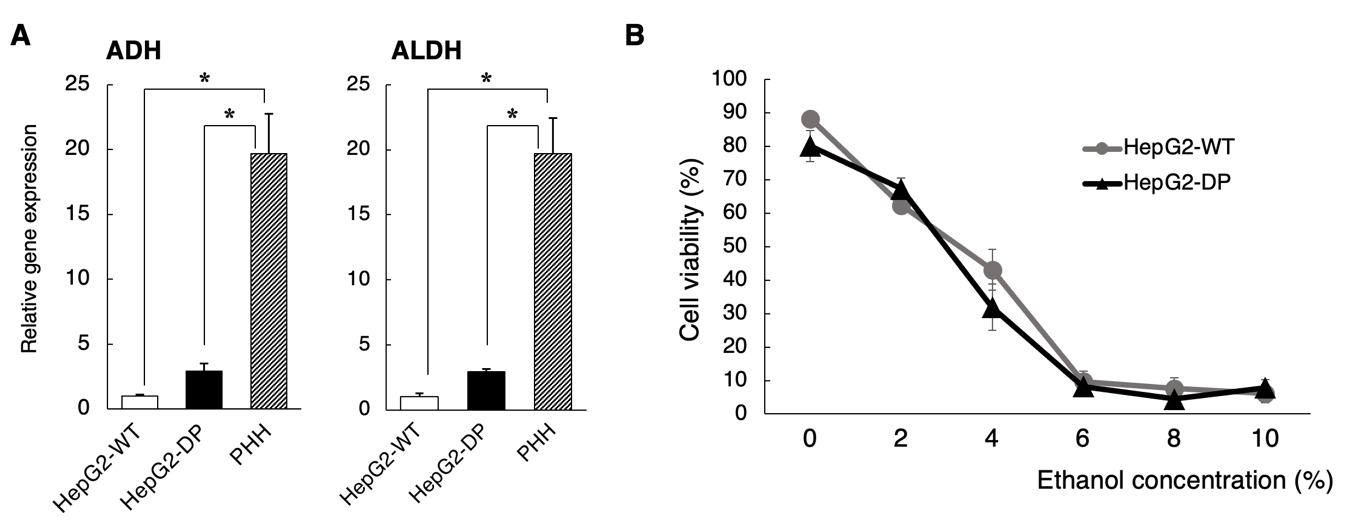
**

**Figure S3.** **Gene expression of alcohol metabolizing enzymes and alcohol toxicity.**

A) Gene expression analysis for alcohol dehydrogenase (ADH) and aldehyde dehydrogenase (ALDH). HepG2-WT cells, HepG2-DP cells, and PHHs were seeded and cultured for 72 h. Reverse transcription polymerase chain reaction (RT-PCR)-examined gene expression levels of cell–cell adhesion factors. The values are the mean ± standard deviation for three samples. Statistical analysis was performed by applying the *t*-test (**p*< 0.05). B) Alcohol toxicity test. Cells were treated with each concentration of ethanol for 24 h and cell viability was measured.

**Figure S4**

**
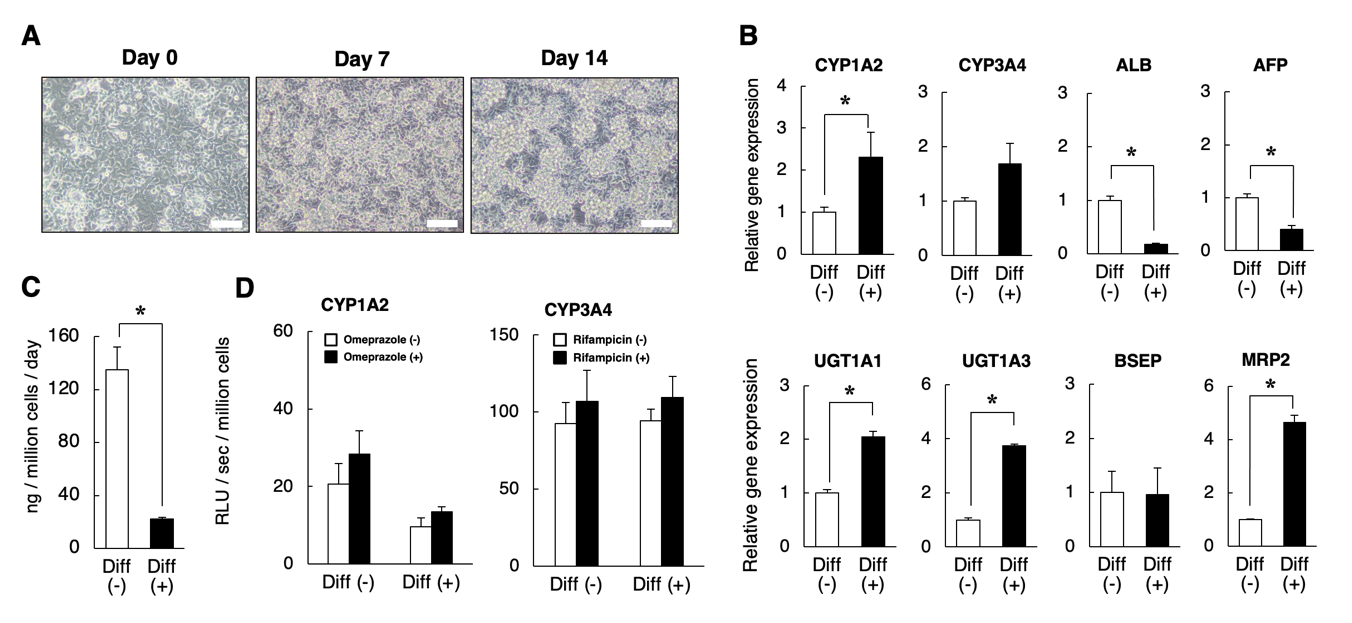
**

**Figure S4.** **Hepatic function in differentiated HepG2-WT cells.**

A) Phase-construct image of HepG2 cells cultured in differentiation medium. The cells were cultured in the differentiation medium for 14 days. Scale bar, 100 μm. B) Gene expression of CYPs, ALB, AFP, bile acid conjugation enzymes, and bile acid transporters. Cells were cultured for 72 h. Reverse transcription polymerase chain reaction–examined gene expression levels of CYP1A2, CYP3A4, ALB, AFP, UGT1A1, UGT1A3, BSEP, and MRP2. Diff (-) indicates the control and Diff (+) indicates the differentiation condition. C) Albumin production of differentiated cells. Albumin production per 24 h was quantified by enzyme-linked immunosorbent assay. D) CYP1A2 and CYP3A4 activity in differentiated cells. Cells were cultured for 48 h and then treated with the indicated inducers for 24 h. CYPs activities were examined using the P450-Glo CYP3A4 assay kit. White bars indicate the no CYP inducer condition and black bars indicate the CYP inducer condition. The values are the mean ± standard deviation for three samples. Statistical analysis was performed by applying the *t*-test (**p*< 0.05).

**Figure S5**


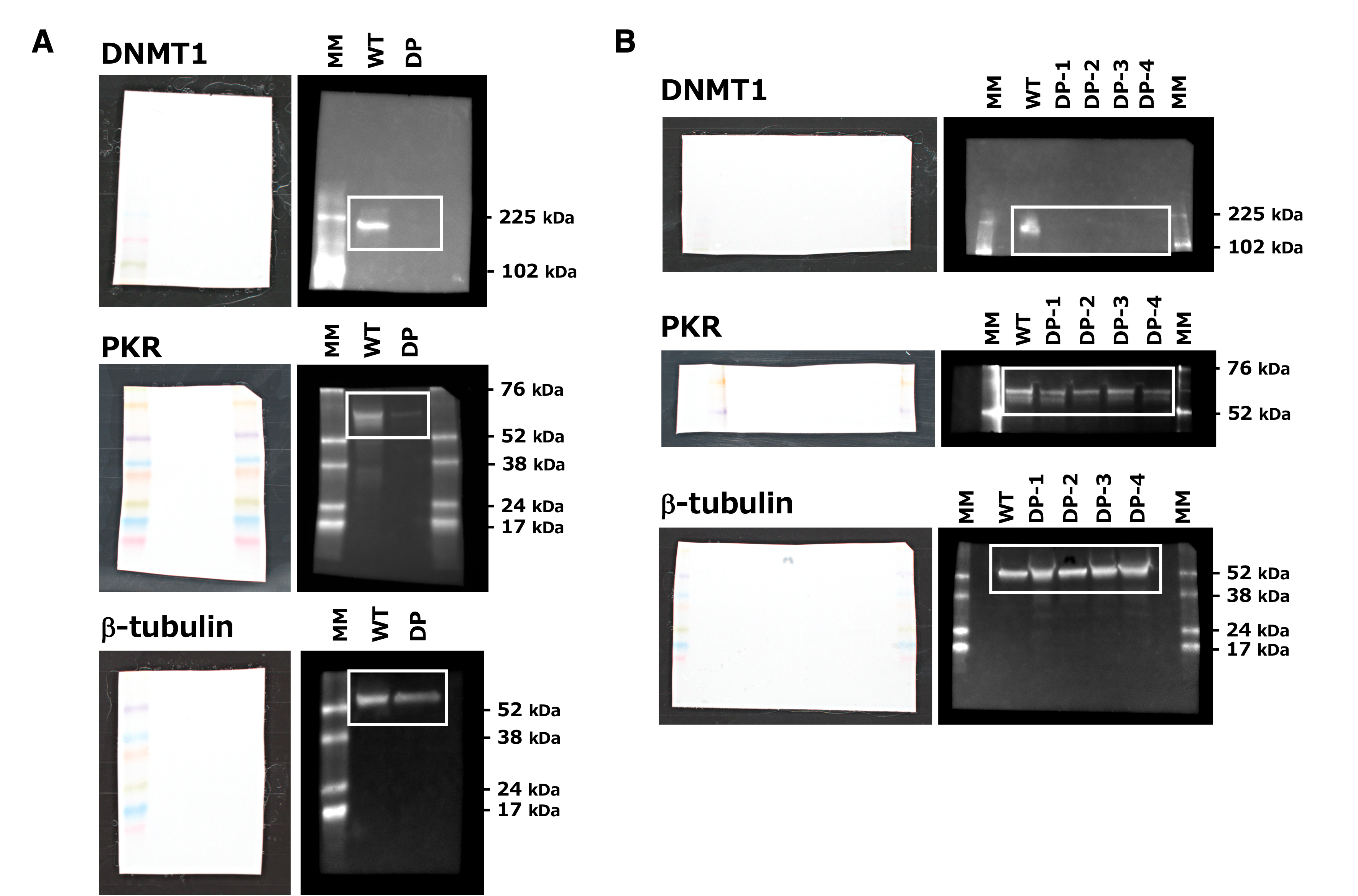


**Figure S5. Original blots of western blotting in Fig.S1A and S1D.**

A) Original blots of Fig. S1A. The boxed area is shown in Fig.S1A. B) Original blots of Fig. S1D. The boxed area is shown in Fig.S1D. MM: molecular weight markers (Full-Range Rainbow™ Molecular Weight Markers, RPN800E).

**Table S1**

**Assay ID of quantitative PCR primers (TaqMan®︎Gene Expression Assay).**

| **Gene** | **Assay ID** |
| --- | --- |
| HNF1A | Hs00167041_m1 |
| HNF1B | Hs01001602_m1 |
| HNF4A | Hs00230853_m1 |
| ONECUT1 | Hs00413554_m1 |
| FOXA1 | Hs04187555_m1 |
| FOXA2 | Hs05036278_s1 |
| FOXA3 | Hs00270130_m1 |
| CYP1A2 | Hs01070371_g1 |
| CYP3A4 | Hs00604506_m1 |
| ALB | Hs00609411_m1 |
| AFP | Hs01040603_m1 |
| UGT1A1 | Hs02511055_s1 |
| UGT1A3 | Hs04194492_g1 |
| ABCC2 | Hs00960489_m1 |
| ABCC11 | Hs00994823_m1 |
| ADH | Hs02383872_s1 |
| ALDH | Hs01116403_m1 |
| CDH1 | Hs01023895_m1 |
| SNAL | Hs00195591_m1 |
| TJP1 | Hs01551876_m1 |
| CTNNA1 | Hs07288000_g1 |
| CDH2 | Hs00983056_m1 |
| GAPDH | Hs02758991_g1 |
